# Fuzzy cognitive mapping with Inuit women: what needs to change to improve cervical cancer screening in Nunavik, northern Quebec?

**DOI:** 10.1186/s12913-020-05399-9

**Published:** 2020-06-11

**Authors:** Elyse Tratt, Ivan Sarmiento, Rachel Gamelin, Jeannie Nayoumealuk, Neil Andersson, Paul Brassard

**Affiliations:** 1 Center for Clinical Epidemiology, Lady Davis Research Institute, Jewish General Hospital, Montreal, Quebec Canada; 2 Department of Family Medicine, CIET-Participatory Research at McGill, Faculty of Medicine, McGill University, Montreal, Quebec Canada; 3 Saturviit Inuit Women’s Association of Nunavik, Kuujjuaq, Quebec Canada; 4 Centro de Investigación de Enfermedades Tropicales, Universidad Autónoma de Guerrero, Acapulco, Mexico; 5 Department of Medicine, McGill University, Montreal, Quebec Canada

**Keywords:** Cervical cancer, Screening, Participatory research, Indigenous health, Inuit, Human papillomavirus

## Abstract

**Background:**

Among Canadian Inuit, cervical cancer incidence and mortality rates are up to three times higher than the Canadian average. Cervical cancer is preventable through regular screening which, in Quebec, is opportunistic and requires physical examination and Papanicolaou (“Pap”) smears. Since Human Papillomavirus (HPV) is the necessary cause of cervical cancer, HPV testing is a plausible screening alternative. HPV testing by self-sampling also addresses several barriers associated with physical examination and access to healthcare. In a participatory research paradigm, we worked with two communities of Nunavik to explore the possible implementation of HPV self-sampling.

**Method:**

Key community stakeholders formed an Advisory Committee to guide direct discussions with Inuit women. We presented available facts around cervical cancer, HPV and the female anatomy, and used Fuzzy Cognitive Mapping to collate women’s views. A thematic analysis summarized data, adding links and weights to represent the relationship of each factor on the outcome: screening for cervical cancer.

**Results:**

According to the 27 Inuit women who participated, the most influential factor in using health services was the cultural awareness of the healthcare provider. A significant barrier to screening was patient lack of information. The principal vector of change – the factor most likely to influence other factors – was the means of communication between the healthcare provider and the patient: visual communication was told to be the most effective.

**Conclusion:**

Fuzzy Cognitive Mapping is a practical tool for discussing possible health actions with stakeholders and to inform future research. The tool offers a visual aid for discussion across cultural and educational differences. It can help to build the partnerships that incorporate community voices into co-design of interventions that are relevant to and aligned with the needs of those who use them.

## Background

The region of Nunavik in northern Quebec is home to some 13,000 people in 14 communities [1], 95% of whom self-identify as Inuit [1]. Cervical cancer incidence and mortality rates among Canadian Inuit are two to three times higher than the Canadian average [2–4]. Prevention through regular screening contributes to early detection and increases the chances for successful treatment of abnormalities before they become cancer [5]. In Quebec, current opportunistic screening uses the Papanicolaou (“Pap”) smear, requiring physical examination [6]. Since cervical cancers are attributable to human papillomavirus (HPV) infection [7], HPV testing offers a plausible alternative for primary screening [8].

Previous research with Inuit women of Nunavik showed HPV testing by dry self-collection of cervicovaginal samples (self-sampling) addresses several barriers associated with physical examination and women’s screening behavior, and it was preferred by 55% of the participating women [9]. This might have been associated with, though not limited to, experience with previous screening through Pap smear. For example, 36.9% reported feelings of embarrassment and 49.4% reported feeling pain at least sometimes [10]. These findings led us to explore how to improve cervical cancer screening uptake, considering the possible future implementation of HPV self-sampling in the region. Our current work with Inuit women in Nunavik seeks to identify the best ways to implement HPV self-sampling as a way to increase access to routine screening, particularly among those under-screened or never-screened.

In a participatory research paradigm [11], we invited Nunavik women to develop their own theory of what needs to change to improve screening in the community. Fuzzy Cognitive Mapping (FCM) is useful in overcoming language barriers, in multicultural contexts, and in settings with educational disparities [12] . It is a flexible technique that uses graphical language of conceptual maps to represent factors contributing to an outcome, and to summarize relationships between those factors [13] [14]. FCM has been extensively used to involve communities in environmental planning [15, 16] and has proven its value in portraying indigenous perspectives on health determinants [17]. The maps are soft models indicating causal relationships [18] and can be used to clarify scenarios [19] or to identify the variables with higher influence in a causal system [20, 21].

An advantage of FCM is the relative computational simplicity of analyzing complex systems, as the maps can incorporate self-pointing loops and cyclic relationships [22]. The maps allow multiple perspectives in a comparable format, so they can contrast and combine different knowledges on a particular topic [23]. This provides formal alternative to amplify the voices of those who are often excluded from framing research issues [24]. We employed FCM to explore barriers and facilitators to screening for cervical cancer by HPV self-sampling and used this to facilitate conversations between Inuit women and Western researchers.

## Methods

### Building partnerships

Following the Canada’s Tri-Council Policy Statement on Ethical Conduct for Research Involving Humans (TCPS2), Chapter 9 on Research Involving the First Nations, Inuit and Métis Peoples of Canada [25], we formed an advisory committee of stakeholders from the Health Center (Professional Services, Public Health), the Regional Health Board (Planning and Programming, Inuit Values and Practices), the regional Inuit Women’s Association, and community representatives. The committee directs and oversees this research through regular communication in person, by phone, and by email with the research team. Two women field researchers with previous experience working in Nunavik facilitated the sessions: a post-graduate student in nursing and a sociology graduate research assistant.

### Engaging Inuit women in conversations

We convened groups of local Inuit women over the age of 25, recruiting participants by word-of-mouth, local radio station, and the communities’ Facebook page. We invited participants to join conversations about cervical cancer screening in a location of mutual convenience, ensuring reasonable privacy and confidentiality. Mapping groups had between one and eight participants, depending on their availability and comfort level. Some who did not speak English or French participated with the help of other women who translated the conversations. After each session, the participants invited other women to participate, allowing us to convene additional sessions based on experienced participants. We offered snacks and refreshments but no monetary compensation; women voluntarily shared their time. We explained the procedure and the intended use of the data, and participants gave verbal consent. The latter was used to address language barriers and literacy gaps within the participants. Verbal consent was also chosen to respect the oral tradition of Inuit culture. We recorded no personal data other than the age of each participant.

**Fig. 1:**
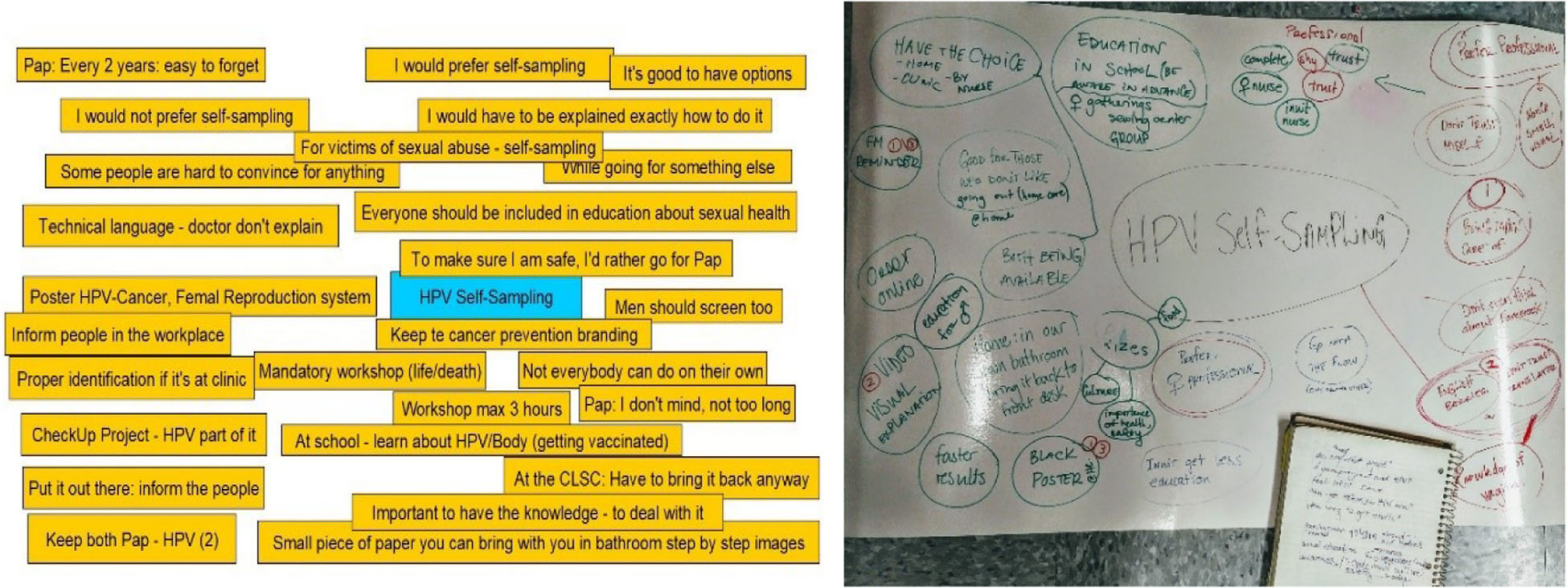
Conceptual Maps

When a screen and projector were available, we used the software yEd [26] to draw the maps in meeting rooms. In other settings, we used a whiteboard and markers. Researchers sat together with the participants around a table or on the floor, facilitating horizontal communication in conversation rather than as an interview [27]. The setup intended to promote a culturally safe environment and exchange for mutual learning [28, 29]. There was no specific discussion guide used or developed for this study and the session facilitators adapted to what was of interest for the participants. The two field researchers presented evidence with visual aids: the female anatomy, the link between cervical cancer and HPV, and the HPV self-sampling technique. They answered all medical questions from the women to ensure every participant understood available facts around cervical cancer screening, before being asked to talk about it. Considering the abundance of questions and the acute interest of participants in evidence surrounding cervical cancer and the self-sampling method, the field researchers spent much more time on this step than initially planned. They then asked participating women what barriers and facilitators they expected to the possible implementation of HPV self-sampling in their community. The conversation unpacked the question into topics about the experience of women: what did they think of the technique? Whether they would use it? Why, or why not? As conversations advanced, the researchers drew the factors women mentioned as conceptual maps (Fig. 1). They also took notes describing the context, the nuances of the conversations, and the relative importance of each factor. At the end of each session, the group reviewed and validated their map.

### Mapping and weighting Women’s knowledge

After completing all sessions, a thematic analysis [30] by the field researchers synthetized information from all cognitive maps into one, reflecting and discussing each theme to preserve as much as possible the meaning and authenticity of all participants’ points of view. The advisory committee then discussed and validated the emerging themes. We linked the main concepts according to reported relationships of causality in relation to the outcome. Each arrow has a direction and a weight illustrating the impact of an element on the other. A positive relationship was when the increment of one factor implied an increment in the landing factor. If the relationship is negative, the increment implied a reduction of the other. Based on the notes of the importance attributed to each relationship by the participating women, the researchers assigned numeric weights between 1 (lower importance) and 5 (higher importance). In most FCM exercises, this step is done by the participants themselves. Because of the time presenting evidence at the beginning of each mapping session, however, the field researchers relied on their notes instead of taking more of the already generous time already given by the participants [31].

## Results

**Fig. 2:**
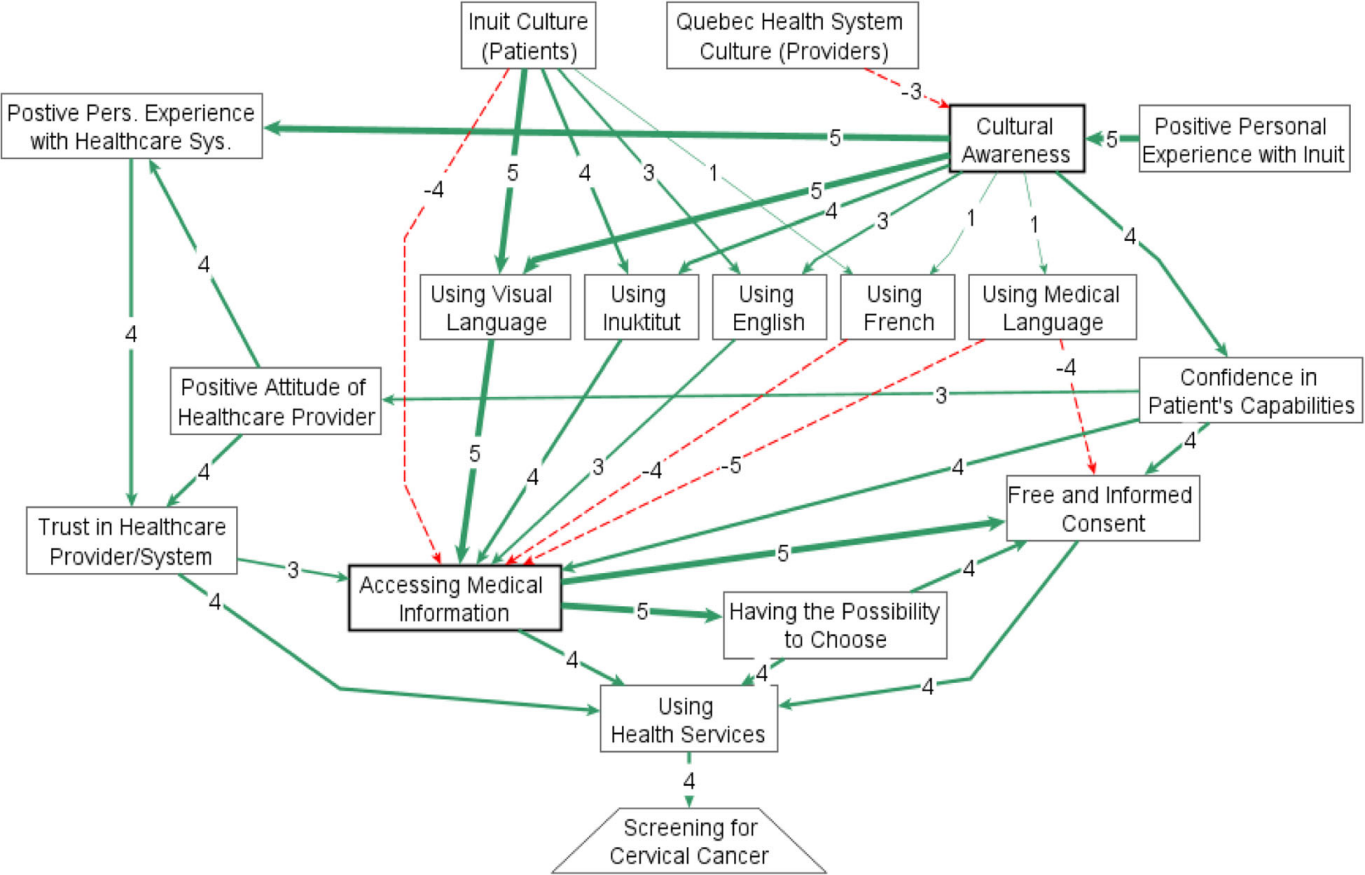
Inuit views of facilitators and barriers to cervical cancer screening – composite results of 10 fuzzy cognitive mapping sessions in Nunavik, Quebec

A total of 27 Inuit women joined 10 meetings. The final map had 18 themes interacting through 35 relationships. In Fig. 2, full lines represent positive relationships and dotted lines, negative relationships. The thickness of the lines is proportional to their weights. The map shows an intercultural context of Inuit patients receiving care from non-Inuit professionals, within the Quebec healthcare system.

We illustrated different trajectories to better understand what makes Inuit women decide to screen for cervical cancer. It allows for an initial estimation of the relative importance of each factor, according to the knowledge of the women who participated. For them, screening for cervical cancer in particular is a consequence of using health services in general. Furthermore, using health services in a preventive manner (screening) is a consequence of access to information.

*Cultural awareness* of healthcare workers was a major facilitator, reflected in positive patient experiences and more use of adequate communication means. *Previous positive experiences* of health personnel in their interaction with Inuit was told to be a factor that highly contributes to increase cultural awareness, countering the negative effect of a healthcare system based on non-Inuit cultural values. An additional path through which more cultural awareness could have positive impacts on the use of health services was mediated by *increased confidence in patients’ capabilities*; thus, allowing a more active role of patient in decision-making. Cultural awareness had an additional strong effect on positive experiences of patients, which would in turn open paths of trust and positive attitudes to use health services and access medical information.

The most important mediator of the system was *access to medical information*. Almost all the factors in the map would connect with increased use of health systems through this factor. The vast majority of participating women expressed the need to get screened after understanding medical facts, including but not limited to the absence of symptoms. The main paths through which more information could have a positive impact on screening for cervical cancer were related to an increased capacity to choose and to participate in decision-making.

All women had different personal preferences for screening methods. Most of them stressed the importance of being inclusive regardless of their own individual preference, as illustrated by this quote from a participant:

*“For me, I am used to getting my Pap, I’m fine with it. But for the younger girls, [or] the shy ones, [or] those who were sexually abused, they might prefer [the HPV self-sampling method].”*

*Means of communication* were important mediators of cultural awareness for access to information and, consequently, better access to available health care. The positive paths opened by adequate communication means contrasted with the negative consequences of the cultural gap faced by Inuit patients. Participants reported the major importance of using visual language, ranking higher than using Inuktitut, and they stressed the communication problems behind using French or medical jargon. This preference for visual communication resonated with our experience using FCM to discuss and summarize conversations with them as, almost without exception, each participant expressed surprise at the coherence and incisiveness of their own analysis. Creating the maps and seeing their knowledge reflected appeared as a validating experience for the participants. We perceived that the exercise itself increased women’s confidence in what they know, what they have to say, and what they can contribute.

## Discussion

The composite map presents the most important factors influencing the use of HPV self-sampling, based on conversations with Inuit women. Participants highlighted a structural dimension of cultural differences that need to be solved before access to health services, and therefore to screening, can happen. Inclusive means of communication were instrumental in each woman accessing sufficient medical information to take the decision to get screened or not, but also to choose *how* to get screened.

FCM provided a reproducible and formal way to summarize a stakeholder-driven theory to identify what needs to change to improve access to health services and consequently improve screening for cervical cancer. In this intercultural context of Inuit patients receiving care from non-Inuit professionals, understanding how Inuit see the issue is central to realistic screening strategies.

Cultural awareness of health personnel allows practitioners to recognize and understand cultural differences as well as the impacts of such differences in health care [32]. The practical implication of this awareness is cultural competence, which aims to make health care services more accessible, acceptable and effective for people from diverse ethno cultural communities [33]. Increased cultural awareness of healthcare providers has shown benefits in patient satisfaction, mutual understanding, adherence to treatment, and knowledge, attitudes, and skills of medical students [34–36]. Health education still lacks adequate training programs to increase the much-needed cultural competency of service providers working in intercultural contexts [37]. The structural gap between Inuit culture and a health system shaped in the Western culture has long been identified as a major source of health inequities for indigenous groups [38, 39]. Despite the benefits of cultural competence, indigenous perspectives demand more critical approaches under the umbrella of cultural safety to promote structural change [40, 41].

A drawback of this exercise using FCM in Nunavik was the difficulty to fully involve participants in the weighting of variables. In addition to time restrictions, cultural differences in the understanding of causality might pose an extra layer of difficulty. More research on this topic could contribute to extend the application of FCM in indigenous contexts.

## Conclusion

Fuzzy Cognitive Mapping is a practical tool for discussing possible health actions with stakeholders and to inform future research. The tool offers a visual aid for discussion across cultural and educational differences. It can help to build the partnerships that incorporate community voices into co-design of interventions that are relevant to and aligned with the needs of those who use them.

## Data Availability

Data sharing is not applicable to this article as no datasets were generated or analysed during the current study.

## Abbreviations

FCM: Fuzzy Cognitive Mapping
HPV: Human papillomavirus
TCPS2: Tri-Council Policy Statement on Ethical Conduct for Research Involving Humans
